# Recovering together: building resiliency in dyads of stroke patients and their caregivers at risk for chronic emotional distress; a feasibility study

**DOI:** 10.1186/s40814-020-00615-z

**Published:** 2020-05-25

**Authors:** Sarah Bannon, Ethan G. Lester, Melissa V. Gates, Jessica McCurley, Ann Lin, Jonathan Rosand, Ana-Maria Vranceanu

**Affiliations:** 1 Integrated Brain Health Clinical and Research Program, Department of Psychiatry, Massachusetts General Hospital, One Bowdoin Square, Suite 100, Boston, MA 02114 USA; 2 Division of General Internal Medicine, Department of Medicine, Massachusetts General Hospital, 100 Cambridge St, Suite 1600, Boston, MA 02114 USA; 3 Henry and Allison McCance Center for Brain Health, Department of Neurology, Massachusetts General Hospital, Boston, MA USA; 4 Neuroscience Intensive Care Unit, Massachusetts General Hospital, Boston, MA USA; 5 Harvard Medical School, Boston, MA USA

**Keywords:** Stroke, Dyads, Patient, Caregivers, Depression, Anxiety, Post traumatic stress, Intervention, Video, Telehealth

## Abstract

**Background:**

A stroke is a sudden, life-altering event with potentially devastating consequences for survivors and their loved ones. Despite advances in endovascular and neurocritical care approaches to stroke treatment and recovery, there remains a considerable unmet need for interventions targeting the emotional impact of stroke for both patients and their informal caregivers. This is important because untreated emotional distress becomes chronic and negatively impacts quality of life in both patients and caregivers. Our team previously used mixed methods to iteratively develop a six-session modular dyadic intervention to prevent chronic emotional distress in patients with stroke and their informal caregivers called “Recovering Together” (RT) using feedback from dyads and the medical team. The aim of the current study is to test the feasibility of recruitment, acceptability of screening and randomization methods, acceptability of RT, satisfaction with RT, feasibility of the assessment process at all time points, and acceptability of outcome measures. Secondarily, we aimed to explore within-treatment effect sizes and change in clinically significant symptoms of depression, anxiety, and post-traumatic stress (PTS). The larger goal was to strengthen methodological rigor before a subsequent efficacy trial.

**Methods:**

We conducted a feasibility randomized controlled trial to evaluate the RT intervention relative to minimally enhanced usual care (MEUC) in stroke patients admitted to a Neurosciences Intensive Care Unit (Neuro-ICU). Dyads were enrolled within 1 week of hospitalization if they met specific eligibility criteria. Assessments were done via paper and pencil at baseline, and electronically via REDCap or over the phone at post-intervention (approximately 6 weeks after baseline), and 3 months later. Assessments included demographics, resiliency intervention targets (mindfulness, coping, self-efficacy, and interpersonal bond), and emotional distress (depression, anxiety, and PTS). Primary outcomes were feasibility and acceptability markers. Secondary outcomes were depression, anxiety, PTS, mindfulness, coping, self-efficacy, and interpersonal bond.

**Results:**

We consented 20 dyads, enrolled 17, and retained 16. Although many patients were missed before we could approach them, very few declined to participate or dropped out once study staff made initial contact. Feasibility of enrollment (87% of eligible dyads enrolled), acceptability of screening, and randomization (all RT dyads retained after randomization) were excellent. Program satisfaction (RT post-test M = 11.33/12 for patients M = 12/12 for caregivers), and adherence to treatment sessions (six of seven RT dyads attending 4/6 sessions) were high. There were no technical difficulties that affected the delivery of the intervention. There was minimal missing data. For both patients and caregivers, participation in RT was generally associated with clinically significant improvement in emotional distress symptoms from baseline to post-test. Participation in MEUC was associated with clinically significant worsening in emotional distress. Although some of the improvement in emotional distress symptoms decreased in the RT group between post-test to 3 months, these changes were not clinically significant. RT was also associated with substantial decrease in frequency of individuals who met criteria for clinically significant symptoms, while the opposite was true for MEUC. There were many lessons that informed current and future research.

**Conclusions:**

This study provided evidence of feasibility and signal of improvement in RT, as well as necessary methodological changes to increase recruitment efficiency before the future hybrid efficacy-effectiveness trial.

**Trial registration:**

NCT02797509.

## Introduction

### Background

Stroke is a highly prevalent public health concern, and the leading cause of death and disability among adults in developed countries [1, 2]. In the USA alone, stroke has an annual incidence of approximately 795,000, and is associated with an estimated 34 billion dollar cost from health care services, medications, and missed days of work [1]. A stroke is often a devastating life-altering event for both patients and their family/friend caregivers [3–5]. Stroke occurs without warning and may result not only in death or profound disability but also substantial emotional trauma for both patients and caregivers [5, 6]. Although advances in endovascular and neurocritical care approaches to stroke treatment and recovery have substantially increase the number of survivors, there remains a need to develop treatments to address the emotional sequelae associated with the stroke and subsequent hospitalization for both patients and their informal caregivers. Rates of clinically significant symptoms of depression and anxiety (30–72% patients; 55–68% caregivers), as well as post-traumatic stress (PTS; 29.6% patients; 20% caregivers) are high [7–16] at hospitalization for an acute brain injury (including a stroke) and predictive of chronic emotional distress at 3- and 6-months later for both patient and caregiver [8–10]. However, there are no evidence-based psychosocial interventions focused on prevention of chronic emotional distress in this population.

Emerging cross-sectional and prospective research, including our own, has shown that patient and caregiver psychosocial factors influence each other and shape each person’s recovery trajectory after a brain injury (such as a stroke), including outcomes of PTS, anxiety, and depression [17–19]. Poor patient mental health is not only directly associated with poor physical recovery but also translates in greater caregiving burden, which negatively impacts caregivers’ mental health [20]. In turn, caregivers’ poorer mental health lowers the quality of care to the patients [20, 21], which impacts patients' physical and mental health [22, 23]. This interdependence between patient and caregiver factors requires consideration of the dyad (e.g., pair of patient and caregiver) in order to fully capture an individual’s level of mental health risk following a stroke.

Dyadic interventions that account for the interdependence between patient and caregiver factors across the recovery trajectory may be the most effective and efficient way to address emotional distress in this high-risk population. This contention is supported by two recent systematic reviews from the American Heart Association [24, 25] which included available psychosocial interventions for stroke patients and caregivers. These reviews highlighted substantial limitations of current interventions for stroke patient and/or caregivers, such as an emphasis on treatment rather than on prevention of emotional distress, a focus on improving either patient or caregiver factors without accounting for their interdependence, and a lack of modular and tailored interventions to account for the heterogeneity of stroke patients' and caregivers' unique challenges and concerns. These systematic reviews recommended the development of dyadic (i.e., both patient and caregiver together) interventions that are tailored to the unique needs of heterogeneous stroke patients and caregivers and aimed at preventing chronic emotional distress in both members of the dyad. Dyadic interventions are also more economical both in terms of interventionist time, as well as overall treatment cost [26–29].

In line with these recommendations, we used a mixed-methods approach to develop Recovering Together (RT)—the first dyadic intervention aimed at *preventing* chronic emotional distress in at-risk stroke dyads. First, we conducted cross-sectional and prospective studies with patients and their primary caregivers at hospitalization in a Neuroscience Intensive Care Unit (Neuro-ICU) and identified key modifiable intervention targets associated with emotional distress (e.g., mindfulness, coping, interpersonal bond, and self-efficacy) [7–10, 16]. Next, we conducted 24 qualitative interviews with stroke dyads followed by three focus groups with nurses [15] to determine dyads’ challenges associated with the stroke and subsequent hospitalization, perceptions of the proposed intervention targets, ways to best teach the proposed resiliency skills, interest in the intervention, and best modality for intervention delivery. We subsequently treated one stroke dyad with Recovering Together [30] and made refinements from lessons learned.

In the present study, we report on a feasibility randomized control trial (RCT) of RT versus a minimally enhanced usual care (MEUC) control group, which followed recommendations for rigorous feasibility testing [31, 32]. The study had two primary aims. First, we measured feasibility and acceptability markers (primary outcomes), including (1) ability to recruit dyads, (2) ability to retain dyads, (3) acceptability of randomization, (4) credibility, and (5) satisfaction. Second, we assessed the signals of within group improvement (baseline to post-test to 3 months follow-up) in emotional distress (e.g., symptoms of depression, anxiety and PTS) and intervention targets (mindfulness, coping, interpersonal bond, and self-efficacy) for patients and caregivers (secondary outcomes) randomized to RT and for those randomized to MEUC.

## Methods

### Design

The current feasibility study was part of a sequential framework for intervention development [33]. Our larger goal was to prepare for a future hybrid efficacy-effectiveness study testing RT’s effect in order to scale, implement, and disseminate this intervention to other Neuro-ICUs in diverse geographical regions across the USA and beyond. This feasibility RCT was conducted in the Neuro-ICU at a major urban medical teaching hospital. We compared the novel RT intervention to a MEUC condition (educational pamphlet). Simple randomization was performed using a random number generator to maintain balance between the groups during random assignment. Participants were not blinded to intervention versus the control. Participants completed questionnaires in person (i.e., during inpatient admission, with staff assistance, as needed) at baseline and either electronically through a link emailed directly to patients and caregivers via the secure web-based data collection platform REDCap [34] or over the phone, at post-test [~ 6 weeks after baseline] and 3-month follow-up. The trial was designed to address specific objectives relating to study design and methodology for the subsequent hybrid efficacy-effectiveness trial and was not designed to determine efficacy [31, 32]. There were no important changes to methods (e.g., eligibility criteria) after the trial commenced. Our Institutional Review Board approved all study procedures prior to study inception.

### Recovering Together intervention

Recovering Together (RT) is a six-session, seven-module (two universal and five specific), skills-based dyadic intervention targeting the prevention of chronic emotional distress in patients and their informal caregivers. RT is informed by elements of traditional cognitive behavioral therapy (CBT; e.g., cognitive restructuring/reappraisals, adaptive thinking), dialectical behavior therapy (DBT; e.g., mindfulness, dialectics, distress tolerance), and principles from trauma-informed care (e.g., impact of the illness/injury, understanding triggers, role and identity changes, meaning making), to address unique needs experienced by each dyads [15]. The intervention directly targets self-efficacy, mindfulness, coping skills, and intimate bonds within dyads, consistent with our prior quantitative and qualitative research [7, 9, 10, 13, 15, 16]. The first two sessions are delivered to all dyads, are conducted with both the patient and caregiver at bedside, and teach the core skills of mindfulness, dialectics, diaphragmatic breathing, and self-care. The following four sessions take place after the patient’s discharge via a secure video platform (i.e., Vidyo software). The patient and caregiver collaborate with the clinician to identify four out of the five available modules that best align with the dyad’s presentation and needs. These sessions build upon and rehearse skills learned in the first two sessions, and teach additional skills and topics selected by dyads [15]. All sessions are approximately 20–30 min and modules can be delivered in any order deemed appropriate/relevant. Table 1 provides session titles and a description of the treatment components.

**Table 1:** Session content for Recovering Together (RT)

Sessions/(modules)	Session content and skills
In the Neuro-ICU, in person at bedside	
1. Coping with the here and now	Deep breathing, mindfulness, staying present (24-h block), meditation and self-care
2. Coping with uncertainty	Sticking with new habits, acknowledging contradictions (dialectics), coping with worry, skills for acceptance and change
After discharge, via secure live video	
3. Adjusting to life after stroke	Challenges adjusting to life after stroke, understanding stressors (self and others), and coping with stress
4. Navigating interpersonal relationships	Relationship role and self-image changes after stroke, skills for acceptance and change, effective communication and interpersonal effectiveness
5. Adherence to rehabilitation regimens	Sticking with your rehabilitation program, making time for self-care, setting SMART goals
6. Fear of stroke recurrence	Mindfulness strategies to cope with fear and worry, using the decision tree for acceptance and change
7. Making meaning from our experiences	Exploring the stroke and post-stroke experience, balancing change and acceptance, mindset for recovery

Modules 1 and 2 were administered to all dyads. Only four out of the remaining five modules were administered to dyads. These four modules were chosen cooperatively by the therapist and dyads

### Minimally enhanced usual care control

Dyads assigned to the minimally enhanced usual care (MEUC) condition received a three page informational pamphlet on the stroke experience and post-stroke recovery for patients and their informal caregivers. Patient-specific information in this pamphlet included types of events defined as strokes, common symptoms after stroke (e.g., fatigue, changes in eat and sleep, changes in thinking, etc.), and suggestions for coping with emotional challenges after stroke (e.g., get regular and physical exercise, rest when you feel fatigued, spend time with friends and family, etc.). Caregiver-specific information included self-care strategies (e.g., finding times to take breaks from caregiving, keeping balance by eating healthy, exercising, and resting, etc.), ways of seeking community support (e.g., support groups, home health aide services, respite care, etc.), and a list of local and national resources related to stroke.

Both RT and MEUC were delivered in addition to usual care. Usual care in the Neuro-ICU included medical care, nurse monitoring, and specialty care services (physical, occupational, and speech-language therapists, respiratory therapy, chaplaincy). Usual care after discharge typically includes specialty care services (e.g., speech, physical therapy) as determined by the medical team.

### Recruitment, screening, and consent

Patients were identified by study staff who checked the electronic medical record for newly admitted stroke patients. Next, study staff approached potential participants, described the study, and completed screening for eligibility. Eligible stroke patients included those (1) over age 18, (2) with demonstrated English fluency and literacy, (3) who suffered an acute stroke (hemorrhagic and ischemic) within maximum 1 week prior to recruitment, (4) who had a caregiver also willing to participate, and (5) medically and cognitively cleared for participation by the medical team. Eligible caregivers included those who: (1) were over age 18, (2) demonstrated English fluency and literacy, and (3) were the primary caregivers of a patient admitted with an acute stroke. Within each dyad, either the patient or caregiver had to screen in for clinically significant symptoms of depression, anxiety, or PTS. This criterion ensures risk for chronic emotional distress within a dyad, based on prior research [35–37]. Dyads were excluded if they were unable or unwilling to participate in in-person and video sessions, or complete follow up measures electronically or over the phone. If dyads elected to participate, study staff gave the dyad physical copies of the treatment manuals/pamphlets (condition dependent), installed the secure Virtual Visit software (i.e., Vidyo) on their electronic device, and scheduled the dyad’s first session with the clinician who subsequently scheduled all sessions with the dyad at the end of each session/module.

### Assessments

Following the informed consent process, dyads completed self-report questionnaires in the Neuro-ICU at the time of study enrollment (baseline), assisted by study staff, as needed. They completed a similar set of questionnaires after the 6-week study period (post-test), and again 3 months after enrollment (3 month).

#### Demographic questionnaires

Patient and caregiver questionnaires assessed age, gender, race/ethnicity, employment status, marital status, educational level, and mental health history (Table 2).

**Table 2:** Baseline characteristics for study participants

	Intervention (RT) *n* = 7	Control (MEUC) *n* = 9	Total *N* = 16
	M(SD), range	M(SD), range	M(SD), range
Patient characteristics			
Age	56.7 (16.9), 35–82	51.7 (18.5), 21–83	53.9 (17.4), 21–83
	*N* (%)	*N* (%)	*N* (%)
Gender-women	4 (57.1)	5 (55.6)	9 (56.3)
Race			
White	6 (85.7)	8 (88.9)	14 (87.5)
Asian	1 (14.3)	1 (11.1)	2 (12.5)
Marital status			
Single, never married	0 (0.0)	2 (22.2)	2 (12.5)
Married/ civil union	5 (71.4)	6 (66.7)	11 (68.8)
Living with partner	0 (0.0)	1 (11.1)	1 (6.3)
Divorced/ Separated	0 (0.0)	0 (0.0)	0 (0.0)
Widowed	2 (28.6)	0 (0.0)	2 (12.5)
Other	0 (0.0)	0 (0.0)	0 (0.0
Work status			
Student (full/ part time)	1 (14.3)	0 (0.0)	1 (6.3)
Unemployed	0 (0.0)	1 (11.1)	1 (6.3)
Retired	2 (28.6)	1 (11.1)	3 (18.8)
Homemaker	0(0.0)	0 (0.0)	0 (0.0)
Employed full-time	3 (42.9)	4 (44.4)	7 (43.8)
Employed part-time	1 (14.3)	1 (11.1)	2 (12.5)
Other	0 (0.0)	2 (22.2)	2 (22.2)
Education			
Some high school (< 12)	1 (14.3)	0 (0.0)	1 (6.3)
High school diploma (12)	1 (14.3)	1 (11.1)	2 (12.5)
Some college/Associates	4 (57.1)	2 (22.2)	6 (37.5)
4-year college	1 (14.3)	3 (33.3)	4 (25.0)
Graduate/ Professional	0 (0.0)	3 (33.3)	3 (18.8)
History of psych conditions			
None	4 (57.1)	7 (77.8)	11 (68.8)
Depression	2 (28.6)	2 (22.2)	4 (25.0)
Anxiety	3 (42.9)	2 (22.2)	5 (31.3)
PTSD	0 (0.0)	0 (0.0)	0 (0.0)
Other	0 (0.0)	0 (0.0)	0 (0.0)
Caregiver characteristics			
Age	49.57 (9.54), 34–63	48.33 (11.94), 27–60	48.88 (10.62), 27–63
	*N* (%)	*N* (%)	*N* (%)
Relationship to patient			
Spouse/partner	5 (71.4)	5 (55.6)	10 (62.5)
Child	2 (28.6)	2 (22.2)	4 (25.0)
Parent	0 (0.0)	1 (11.1)	1 (6.3)
Sibling	0 (0.0)	1 (11.1)	1 (6.3)
Gender-women	5 (71.4)	7 (77.8)	12 (75.0)
Race			
White	6 (85.7)	7 (77.8)	13 (81.3)
Asian	1 (14.3)	1 (11.1)	2 (12.5)
More than 1 race	0 (0.0)	1 (11.1)	1 (6.3)
Work status			
Unemployed	0 (0.0)	0 (0.0)	0 (0.0)
Retired	0 (0.0)	0 (0.0)	0 (0.0)
Homemaker	1 (14.3)	0 (0.0)	1 (6.3)
Employed full-time	5 (71.4)	6 (66.7)	11 (68.8)
Employed part-time	1 (14.3)	0 (0.0)	1 (6.3)
Other	0 (0.0)	3 (33.3)	3 (18.8)
Education			
Less than high school (< 12)	0 (0.0)	0 (0.0)	0 (0.0)
High school diploma (12)	2 (28.6)	0 (0.0)	2 (12.5)
Some college/ Associates	1 (14.3)	2 (22.2)	3 (18.8)
4-year college	4 (57.1)	6 (66.7)	10 (62.5)
Graduate/ Professional	0 (0.0)	1 (11.1)	1 (6.3)
History of psych conditions			
None	3 (42.9)	5 (55.6)	8 (50.0)
Depression	3 (42.9)	2 (22.2)	5 (31.3)
Anxiety	3 (42.9)	2 (22.2)	5 (31.3)
PTSD	2 (28.6)	0 (0.0)	2 (12.5)
Other	0 (0.0)	0 (0.0)	0 (0.0)

The Hospital Depression and Anxiety Scale (HADS) [38] is a widely used, reliable, and valid measure for symptoms of depression and anxiety. It has 14 items answered on a 4-point Likert scale from 0 “*not at all*” or “*very rarely*” to 3 “*all of the time*” or “*very often*.” Scores for depression and anxiety are calculated separately by summing the items for the respective subscales (each including seven items). Higher scores indicate higher symptom severity. Scores equivalent or greater than 8 represent clinically significant symptoms of anxiety or depression. A review of 15 studies reported average internal reliability for the HADS; internal reliability ranged from questionable to excellent for both the anxiety (HADS-A; mean α = 82; range .67 to .90) and depression (HADS-D; mean α = 83; range .68 to .93) subscales [39]. In the current sample, internal reliability was good for patients (α = .86) and was excellent for caregivers (α = .92) for the HADS-A, and similar across time points. For the HADS-D, internal reliability was good for caregivers (α = .82) and questionable for patients (α = .66), though internal reliability improved at post-test and the 3-month follow up (α = .71 and .75). Minimally important clinical differences (MCIDs) for HADS subscales are between 1.5 and 1.7 [40, 41].

The PTSD Checklist-Civilian Version (PCL-C) [42] is a valid and reliable measure of post-traumatic stress (PTS) symptom severity. There are 17-items scored on a 5-point Likert scale ranging from 1 “*not at all”* to 5 “*extremely”.* Total severity scores, as well as clinically significant, are generated by summing all items. A review of 135 studies including the PCL-C indicated that the measure demonstrated excellent internal consistency across studies (α = 93) [43]. Higher PCL-C scores suggest worse symptom severity. The PCL-C had good internal reliability for patients (α = .86) and was excellent for caregivers (α = .92) in our sample. Clinically significant symptoms are determined by following an algorithm based on Diagnostic and Statistical Manual for Mental Disorders (DSM) criteria being met (B-D) with symptoms endorsement in the moderate or above range. A 10-point improvement in symptoms is considered clinically meaningful [44].

The General Self-Efficacy Scale (GSE) [45] is a 10-item measure of ability to resourcefully manage challenging situations. Items are scored on a 4-point Likert scale ranging from 1 “*not at all true”* to 4 “*exactly true*.” Total scores are determined by summing all items together. Higher scores depict higher perceived resourcefulness. Internal reliability was good for patients (α = .89) and for caregivers (α = .85) at baseline and similar at later time points, consistent with previous studies involving the GSE [46].

The Measure of Current Status Part A (MOCS-A) [47] is a measure validated in medically-ill populations that assesses an individual’s ability to employ various coping skills, including relaxation techniques, acknowledgment of stress and tension, ability to be assertive, and altering negative thought patterns, as a responsive to daily life stressors. The 13-items are answered on a 5-point Likert scale with responses ranging from 0 “*I cannot do this at all*” to 4 “*I can do this extremely well*.” Total coping scores are generated by summing all items. Higher scores suggest more efficient coping techniques. In the current sample, internal reliability was acceptable for patients (α = .78) and excellent (α = .95 or higher) for caregivers in the sample, which is similar to the coefficients reported in published studies of NICU caregivers (α = 0.91) [13].

The Cognitive and Affective Mindfulness Scale-Revised (CAMS) [48] is a valid and reliable measure of the extent to which individuals experience thoughts and feelings within the present moment. The 12-item measure is scored using a 4-point Likert scale from 1 “*rarely or not at all*” to 4 “*almost always*.” Total mindfulness scores are computed by summing all the items, with greater scores suggesting greater mindfulness. The CAMS-R demonstrates poor to good internal reliability (αs from .61 to .85) across studies [49, 50]. In the present sample, the CAMS-R demonstrated good reliability for caregivers (α = .86), and questionable internal reliability for patients (α = .54) at baseline, though improved to excellent at post-test and good at 3 months (α = .90 and .87).

The Intimate Bond Measure (IBM) [51] is a measure of participants’ perception of the quality of their interaction with their caregiver or patient, specifically assessing domains of control by one’s partners and perceived care. The 24-items are scored on a 4-point Likert scale with responses from 0 “*not at all true*” to 3 “*very true*.” Items about partner’s perceived control are reverse scored and summed with the remaining care items. Higher scores suggest higher perceived care and lower partner control. Previous studies have found internal reliability coefficients that ranged from questionable to good (α from 0.68 to 0.83) [52]. In this sample, the IBM showed poor internal reliability for patients (α = .54), though improved to questionable at post-test (α = .64) and good (α = .83) at 3 months. Internal reliability was good for caregivers (α = .85) at baseline and similar across time points.

At pre-test, participants completed the Credibility and Expectancy Questionnaire (CEQ) [53], a 6-item measure of treatment expectancy and rationale credibility for clinical outcome studies. The measure has two subscales that demonstrate differential predictive validity among medical populations [54] (1) cognitively based credibility and (2) affectively based expectancy. Possible scores range from 3 to 27 for both the credibility and the expectancy subscales, with higher scores indicating higher perceived credibility/expectancy. In the validation study, the CEQ had a total standardized alpha coefficient (both factors) of .85. In the present sample, the credibility scale demonstrated excellent internal reliability for patients (α = .90) and caregivers (α = .91). The expectancy scale demonstrated good internal reliability for patients (α = .87) and acceptable reliability for caregivers (α = .74).

At post-test, participants completed the Client Satisfaction Questionnaire (CSQ-3) [55, 56], a 3-item measure of satisfaction with the program. The CSQ-3 has been used to measure psychotherapy treatment satisfaction among individuals with depression [57], chronic pain [58], and other physical conditions [59]. Items are scored on a 4-point Likert scale, with higher scores indicating greater satisfaction with delivery of care, satisfaction with clinician, and overall program satisfaction. Internal reliability in this sample was excellent for both patients (α = .90) and for caregivers (α = .95), and similar to those reported across studies of client satisfaction [60].

There were no changes to assessment or measurement procedures after the trial commenced.

### Feasibility and acceptability

Feasibility of recruitment was assessed by determining the number of patients approached in the Neuro-ICU who agreed to participate (% screened/% enrolled).

Acceptability of randomization and procedures was determined by measuring the number of participants who elected to discontinue after they learned their randomization status.

Acceptability of study procedures was determined by the number of participants lost to follow-up (no post-test and 3-month follow-up) in both RT and MEUC.

Adherence to treatment was determined based on the number of sessions attended by participants in RT condition. Dyads were considered adherent if they attended at least four out of six RT sessions.

Feasibility of quantitative measures was deemed acceptable if no questionnaires were missing in full in more than 25% of participants and if reliability was higher than .70 for each questionnaire individually.

### Randomization and allocation concealment

Participants were randomized 1:1 to RT or MEUC (simple randomization). Patient baseline materials contained sealed envelopes. Envelopes were opened by study clinicians after dyads completed the recruitment and screening process, consented to study participation, and completed the baseline measures. Because we compared RT with a minimally enhanced control condition, neither the dyad nor the therapist was blind to condition.

### Identification of study limitation to inform the future trial

The study principal investigator (PI) maintained a constant dialog with the study staff who collected data, the study therapists, Neuro-ICU nurses and medical staff, as well as patients and their caregivers. Bi-weekly team meetings with nursing staff were also held to discuss feasibility issues. The study team documented issues related to recruitment, retention, assessments, and intervention delivery throughout the study to identify and remedy any limitations that were not previously considered. No changes were made to session content or treatment delivery. Finally, we monitored any adverse events in both intervention groups, none of which were observed.

### Data analyses

The current study was a feasibility trial designed to inform a hybrid efficacy-effectiveness trial [31, 32]; as such, it was not designed to detect a treatment effect [61, 62]. Feasibility trials primarily report descriptive statistics on variables, as well as information on recruitment, acceptability of screening, randomization, and quantitative assessments. For the active treatment condition (RT), we report treatment satisfaction and perceived credibility. Data were examined within-treatment and -subject (patients and caregivers separately) in RT and MEUC. First, we present descriptive statistics (means and standard deviations) for the outcome variables at each time point. Next, we present within-subjects changes and effect sizes for improvement from baseline to post-test and from post-test to 3 months using Cohen’s *d* separately for patients and caregivers, and by treatment group (RT vs. control). We specify whether these improvements are above the MCIDs for depression, anxiety, and PTS. Finally, we report frequency of patients and caregivers with clinically significant symptoms of depression, anxiety, and PTS at all time points. There were no interim analyses or stopping rules for this feasibility study.

## Results

### Sample

Baseline participant characteristics are presented in Table 2 for patients and caregivers, separately for the RT and MEUC. Patients were gender balanced and in majority white and educated. Conditions were comparable in terms of age and racial and ethnic distribution.

### Feasibility of recruitment

**Fig. 1:**
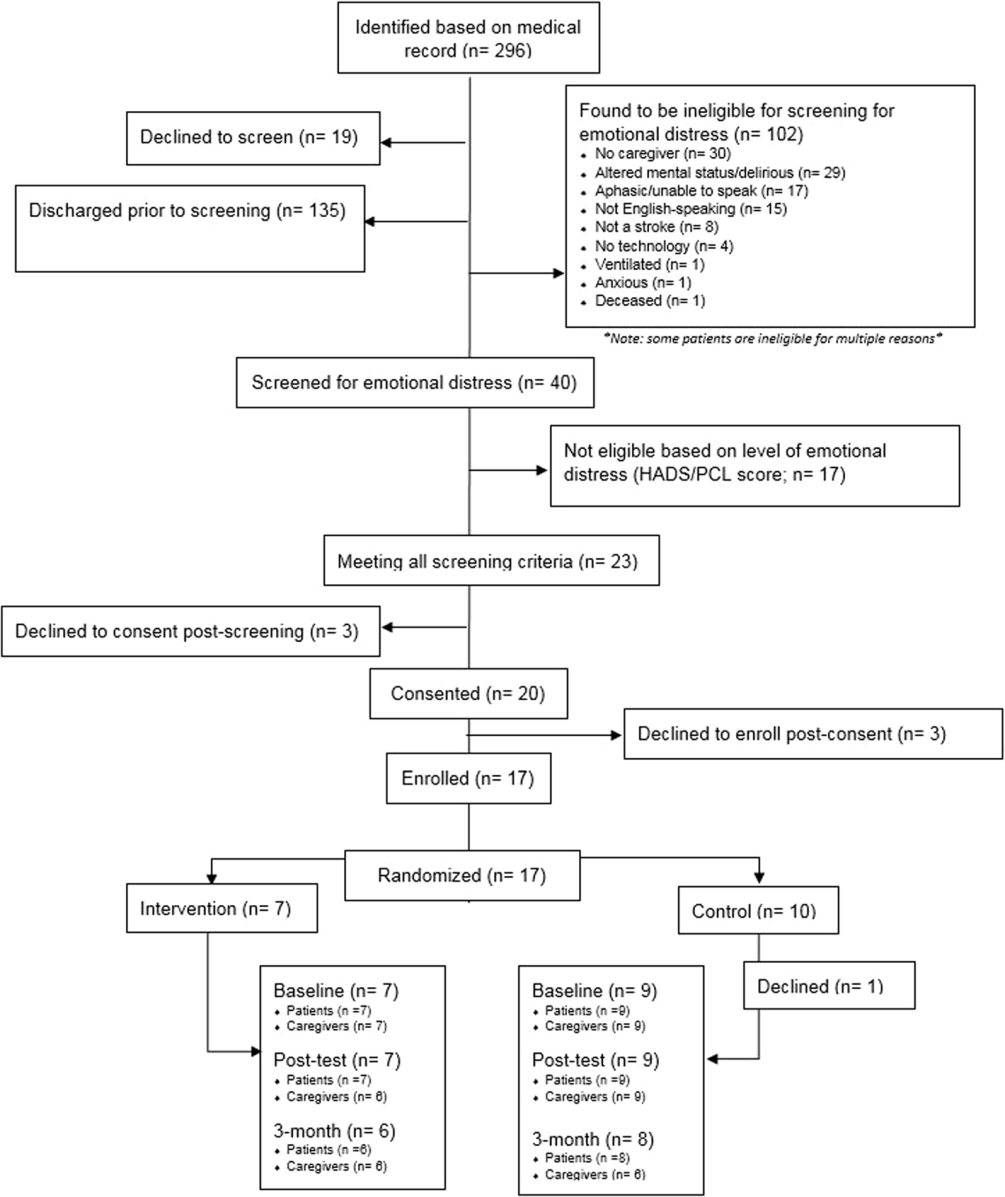
CONSORT

We report the number of individuals referred for screening, approached for further screening, and consented (see Fig. 1 for a CONSORT diagram of the study). Over the course of 1 year, a total of 296 new stroke patients hospitalized in the Neuro-ICU were identified based on the medical record. Of those, 19 declined screening, 135 were discharged before they could be approached by the study team for screening, and 102 potential dyads did not meet initial screening criteria (one through five), in majority due to altered mental status. The remaining 40 dyads who met these initial criteria were further screened for emotional distress using the HADS and PCL. Of these 40, 23 dyads (58%) met criteria of risk for chronic emotional distress by having at least one person (patient or caregiver) that endorsed clinically significant symptoms of either depression, anxiety, or PTS. Of these 23 dyads, three (13%) declined to consent after screening. Twenty dyads completed the informed consent process. Three of the 20 consented dyads (15%) declined to enroll after the informed consent process. The remaining 17 dyads were randomized seven to intervention and ten in control. One dyad in the control condition dropped out of the study after enrollment and randomization procedures, but prior to the completion of baseline measures, citing concerns about finishing study procedures prior to discharge (see Fig. 1).

### Acceptability of randomization

The CONSORT diagram (Fig. 1) shows the flow of participants in the study. Acceptability of randomization was excellent, and no RT dyads refused to participate in the study after randomization to condition.

### Acceptability of study procedures

Among RT dyads, acceptability of study procedures was high. All dyads provided post-test data (*n* = 7; 100%), and all but one dyad (*n* = 6; 85%) provided 3-month follow-up. All dyads who completed baseline measures in the MEUC condition provided post-test data (*n* = 9; 100%), and all but one dyad (*n* = 8; 88.9%) provided 3-month follow-up.

Treatment adherence was good, with four of the seven dyads in RT attending 6/6 sessions, two dyads attending 4/6 sessions, and one dyad failing to attend any sessions because the caregiver lived in a different state, worked full time, and could not travel to the Neuro-ICU during regular hours and study staff was unable to install the video software prior to discharge.

### Feasibility of quantitative measures

Feasibility of quantitative measures was high. All participants in both treatment conditions completed baseline measures and there was minimal missing data. One caregiver in the RT condition and one dyad (patient and caregiver) in the MEUC condition failed to complete post-test measures. Completion of the 3-month measures was also high, and 14 of the 17 dyads completed all quantitative measures (six RT, eight MEUC). Of the participants that completed questionnaires, no measure/inventory was missing in full. For caregivers, internal consistency of measures was high for all measures. For patients, internal reliability was acceptable to excellent in most measures except the IBM, and the HADS-D and CAMS-R, which both improved after baseline and had similar baseline coefficients to those reported in previous studies [39].

#### Treatment credibility and expectancy

Average credibility and expectancy ratings were high for both RT and MEUC patients and caregivers (Table 3).

**Table 3:** Unadjusted means, standard deviations, ranges, and effect sizes for within group tests (patient outcomes)

	Baseline	Post-Intervention	3-month follow-up	Baseline-post effect	Post-3-month effect	Baseline-3-month effect
	*M*(*SD*), Range	*M*(*SD*), Range	*M*(*SD*), Range	Cohen’s *D*	Cohen’s *D*	Cohen’s *D*
Patient outcome						
Depression						
(HADS) RT	4.00 (2.82), 1–8	2.71 (3.35), 0–8	3.00 (3.89), 0–10	− .41	.08	− .29
MEUC	6.31 (4.19), 1–14	10.11 (3.52), 4–16	8.63 (2.88), 5–12	.98	− .46	.64
Anxiety						
(HADS) RT	9.57 (4.89), 3–17	4.33 (3.27), 1–10	4.50 (2.59), 0–7	− 1.25	.06	− 1.29
MEUC	10.24 (4.89), 2–18	12.67 (5.94), 2–19	9.88 (6.47), 2–18	.44	− .45	− .04
Post-traumatic stress						
(PCL-C) RT	36.14 (16.54), 17–61	24.57 (10.61), 17–48	28.50 (11.42), 18–48	− .83	.36	− .53
MEUC	37.33 (7.81), 27-47	43.75 (10.61), 26–57	38.88 (11.82), 20–56	.68	− .43	.15
Self-efficacy						
(GSE) RT	32.57 (5.06), 26–39	34.71 (6.16), 23–40	33.67 (5.68), 26–40	.38	− .18	.20
MEUC	28.78 (6.48), 20–40	26.78 (5.85), 19–39	28.38 (6.30), 18–40	− .32	.26	− .06
Coping skills						
(MOCS-A) RT	30.60 (6.58), 19–35	37.67 (12.03), 17–52	37.40 (8.02), 27–49	.72	− .02	.92
MEUC	26.44 (9.06), 14–38	23.00 (11.75), 10–48	26.14 (12.88), 12–47	− .32	.25	− .02
Mindfulness						
(CAMS) RT	32.29 (4.11), 28–39	34.60 (9.97), 23–44	39.17 (6.77), 30–47	.30	.54	1.22
MEUC	31.56 (5.27), 24–39	27.89 (8.74), 19–45	29.75 (11.77), 15–47	− .51	.18	− .20
Relationship quality						
(IBM) RT	37.57 (4.54), 31–45	44.17 (9.97), 36–60	39.00 (7.48), 31–53	.85	− .59	.23
MEUC	31.56 (5.27), 35–52	40.63 (10.56), 25–56	34.43 (8.00), 22–47	1.09	− .66	.42
Treatment credibility						
(CEQ) RT	22.76 (7.42), 6–27	N/A	N/A	N/A	N/A	N/A
MEUC	19.30 (4.59), 12.60–25.20	N/A	N/A	N/A	N/A	N/A
Treatment expectancy						
(CEQ) RT	23.79 (8.38), 5.4–28.80	N/A	N/A	N/A	N/A	N/A
MEUC	22.28(3.69), 15.30–26.10	N/A	N/A	N/A	N/A	N/A
Treatment satisfaction						
(CSQ-3) RT	N/A	11.33 (1.03), 10–12	N/A	N/A	N/A	N/A
MEUC	N/A	9.00 (2.31), 5–12	N/A	N/A	N/A	N/A
Caregiver outcome						
Depression						
(HADS) RT	3.86 (3.72), 0–8	2.14 (1.07), 0–3	3.60 (2.19), 1–7	− .63	.85	.09
MEUC	3.56 (3.97), 0–11	6.25 (3.62), 0–11	6.17 (4.83), 1–13	.71	− .02	− .59
Anxiety						
(HADS) RT	10.14 (5.61), 3–19	6.83 (1.47), 5–8	7.80 (1.92), 5–10	− .81	.57	.56
MEUC	8.67 (4.90), 1–15	11.00 (4.72), 4–18	9.17 (3.55), 4–13	.48	− .32	− .12
Post-traumatic stress						
(PCL-C) RT	39.00 (19.05), 17–67	25.33 (5.43), 17–32	27.50 (7.74), 22–42	− .98	.32	.79
MEUC	30.78 (7.29), 21–41	35.86 (13.98), 23–59	38.00 (13.24), 23–53	.46	.22	− .68
Self-efficacy						
(GSE) RT	33.57 (2.51), 28–39	33.33 (4.18), 29–40	32.20 (4.60), 26–38	− .07	− .26	− .37
MEUC	32.88 (3.27), 29–38	30.22 (4.79), 22–37	30.67 (4.97), 22–37	− .65	.09	− .53
Coping skills						
(MOCS-A) RT	32.17 (16.24), 7–52	38.83 (7.68), 31–52	33.25 (5.38), 25–38	.52	− .55	.09
MEUC	32.13 (7.40), 21–41	24.38 (10.27), 8–39	27.33 (7.58), 18–40	− .87	.33	− .64
Mindfulness						
(CAMS) RT	36.00 (8.33), 23–45	40.33 (4.76), 34–48	37.00 (6.90), 26–46	.64	− .57	.13
MEUC	36.86 (5.37), 30–44	31.67 (7.58), 19–42	31.67 (5.50), 25–38	− .79	.00	− .95
Relationship quality						
(IBM) RT	39.58 (2.51), 35–42	38.67 (2.80), 36–43	39.40 (4.77), 34–47	− .34	.19	− .04
MEUC	40.50 (9.65), 31–61	36.75 (14.16), 16–58	36.00 (8.81), 25–48	− .31	− .01	− .49
Treatment credibility						
(CEQ) RT	22.68 (4.56), 17.10–27.00	N/A	N/A	N/A	N/A	N/A
MEUC	20.10 (4.57), 11.70–27.00	N/A	N/A	N/A	N/A	N/A
Treatment expectancy						
(CEQ) RT	21.90 (6.25), 11.70–28.80	N/A	N/A	N/A	N/A	N/A
MEUC	24.60 (2.25), 21.60–27.00	N/A	N/A	N/A	N/A	N/A
Treatment satisfaction						
(CSQ-3) RT	N/A	12.00 (0.00), 12–12	N/A	N/A	N/A	N/A
MEUC	N/A	9.00 (1.73), 7–12	N/A	N/A	N/A	N/A

#### Satisfaction with program

Neither patients nor therapists reported substantial challenges with the video platform and were satisfied with the live, remote delivery using their computers, tablets, or phones. Many patients and caregivers expressed enthusiasm and gratitude for the video platform, which did not require travel to the hospital in-person. Treatment satisfaction (CSQ-3) was rated at post-test and was high for both conditions, though higher for dyads in the RT condition (Table 3).

### Means, standard deviations, and ranges

Means, standard deviations, and ranges for all study outcomes at each time point are depicted for patients and their informal caregivers, separately for RT or MEUC in Table 3. In addition, percentages of patients and caregiver within RT and MEUC who reported clinically significant symptoms of depression, anxiety, and PTS at all time points are presented in Table 4.

**Table 4:**
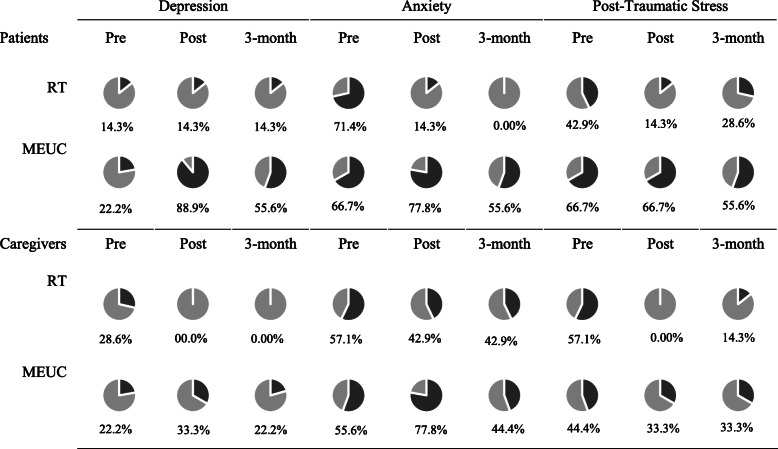
Percentage of participants meeting criteria for emotional disorders

### Within group changes in outcomes

Participation in RT was associated with baseline to post-test *decrease* in symptoms of depression, anxiety, and PTS (medium to large effect sizes) in patients *(d* = − .41, − 1.25, and − .83, respectively) and caregivers *(d* = − .63, − .81, and − .98). Except for patient depression symptoms, changes were all above established MICDs for dyads in RT. Receipt of MEUC was associated with clinically significant *increases* in symptoms of depression, anxiety, and PTS (medium to large effect sizes) for patients (*d* = .98, .44, and .68) from baseline to post-test. For caregivers receiving MEUC, baseline to post-test increases in symptoms of depression, anxiety, and PTS were also observed (*d* = .71, .48, and .46), and increases in depression symptoms exceeded MCID values. Gains in resiliency variables, such as self-efficacy, mindfulness, and perceived coping, demonstrated small to large effect sizes (*d* = .07–.85) in RT but not MEUC dyads from baseline to post-test (see Table 3).

Within-subject effect sizes for RT between post-test and 3 months showed a slight increase in symptoms (small to medium effect sizes) of depression, anxiety, and PTS for patients (*d* = .08, .06, and .36, respectively). These increases, however, were not clinically significant per MCID; 3 months mean scores of emotional distress remained lower than at baseline. Larger increases in symptoms (medium to large effect sizes) were observed for caregivers in RT between post-test and 3 months (*d* = .85, .57, and .32), though all increases were not clinically significant, and mean scores at 3 months remained lower than at baseline. Within subject effect sizes for MEUC from post-test to 3 months indicated decreases in symptoms (medium effect sizes) for depression, anxiety, and PTS, for patients (*d* = .46, .45, and .43), though there were not clinically significant and mean scores at the 3-month follow-up remained higher than baseline. Caregivers in MEUC demonstrated slight decreases in depression and anxiety symptoms from post-test to 3 months (small to medium effect sizes; *d* = .02, .32) and slight increases in PTS symptoms (*d* = .22) but all were not clinically meaningful. All scores at the 3-month follow-up remained higher than baseline. Overall, we observed a general trend for decrease in emotional distress from baseline through 3 months for RT dyads, and an increase in emotional distress for MEUC dyads in the same time frame.

### Clinically significant symptoms

At baseline, a majority of patients and caregivers screened positive for anxiety and PTS, with fewer screening for depression (Table 4). At post-intervention assessment, the rates of clinically significant anxiety and PTS for patients and caregivers in RT were substantially lower. Improvements in depression symptoms were seen in RT caregivers at post-intervention that persisted through the 3-month follow up, though RT patients' rates remained stable (14.3%) throughout the study assessment period. In MEUC dyads, we observed increases in rates of clinically significant symptoms of depression, anxiety, and PTS at post-intervention and 3 months.

## Discussion

We conducted a randomized controlled feasibility trial of a dyadic resiliency intervention (Recovering Together; RT) versus a minimally enhanced usual care (MEUC) comparison, with the goal of informing the design and conduct of a future hybrid efficacy-effectiveness study aimed at preventing chronic emotional distress in stroke patients and their informal caregivers. Recruitment was challenging and many dyads were missed before study staff was able to approach. However, once recruited and randomized, dyads in the RT condition attended most study sessions and nearly all dyads in both conditions completed post-test and follow-up questionnaires. Attrition was better than in other clinical protocols of stroke patients and their informal caregivers [25]. There were no technical issues associated with the delivery of RT via secure live video. The feasibility and acceptability of study procedures was also high, though some questionnaires demonstrated low internal reliability for patients at baseline. Additional measures of study feasibility such as credibility, expectancy, and satisfaction were also high. These results are encouraging and show that patients with stroke and their caregivers are generally receptive to psychosocial interventions targeting prevention of chronic emotional distress.

Exploration of within-group effect sizes demonstrated improvement (decreased symptoms) in all emotional distress outcomes between baseline and post-test (moderate to large effect sizes) in RT, with improvements over MCIDs for anxiety and PTS in patients and for depression, anxiety, and PTS in caregivers. Symptoms increased slightly from post-test to 3 months for RT dyads, though none of the changes were clinically significant. In contrast, patients in the MEUC condition demonstrated clinically significant increases in symptoms in all emotional distress outcomes between baseline and post-test (medium to large effect sizes) and caregivers (above MCID for depression symptoms). Symptoms slightly decreased from post-test to 3 months for MEUC dyads, though all decreases were below MCID values. Over the course of 3 months (baseline to 3 months), we observed a general improvement in emotional distress for dyads randomized to RT and a general deterioration in symptoms for those in MEUC.

Across all dyads, clinically significant symptoms of anxiety and PTS symptoms were more common than symptoms of depression. We observed a substantial decrease in frequency of both patients and caregivers with clinically significant emotional distress from baseline to both post-intervention and 3 months for those randomized to RT. Of note, in the RT condition, the percentage of patients exhibiting clinically significant depression symptoms at baseline was low (14.3; one patient) and remained stable; rates of clinically significant depression symptoms were higher in RT caregivers (28.6%; two caregivers) and improved to 0% at post-test and 3 months. In MEUC, rates of clinically significant depression, anxiety, and PTS remained stable or increased. Taken together, these findings show evidence of potential benefit from RT in reducing risk for chronic anxiety and PTS in both stroke patients and caregivers, but no evidence for improvement in depression. This may be a function of the small sample size with only seven cases of depression, as well as stroke-related biochemical changes in depression, lack of skills in RT to properly target depressive symptoms, or a combination of these factors. The substantial deterioration in symptoms observed in MEUC is consistent with prior studies which show that emotional distress at hospitalization predicts future emotional distress in both patients and caregivers [10, 16]. These findings underscore the need for proactive intervention for prevention of emotional sequelae that occur in the wake of stroke for dyads [30].

This feasibility trial allowed us to study and understand challenges of conducting research in a high stress and busy Neuro-ICU setting. There were many lessons learned (Table 5) which will be implemented in the future trial in order to streamline recruitment. One of the most challenging aspects of the study was reaching the substantial volume of patients with stroke to evaluate eligibility. The Neuro-ICU admission rate is high, and many patients are hospitalized for only a few days, during which their exhaustion and medical acuity is high. It was difficult for study staff to determine, based on the medical record alone, which dyads would be likely to meet study criteria including medical clearance to participate. Although we screened as many dyads as possible, we likely missed many potentially eligible dyads while screening many who ended up unable to participate due to impaired medical or cognitive presentation. Taken together, these findings highlight the need to work closely with the Neuro-ICU medical and nursing team to streamline recruitment so that we can approached only those dyads who are medically and cognitively cleared for participation by the medical team.

**Table 5:** Problems identified in the present study and potential solution

Problem	Solution
1. Unable to approach dyad for study prior to Neuro-ICU discharge.	1. Research staff are available in the Neuro-ICU daily.
2. Approached patients who are ineligible due to altered mental status.	2. Develop a collaboration with the nursing staff so that they can refer for participation patients who are mentally able to enroll. Approach only this select number of patients.
3. Declined to consent after screening.	3. Perform screening, consent, assessments and first session in the same day.
4. Declined to enroll post consent before randomization.	4. Perform consent, assessment, and first session in the same day.
5. Caregiver unable to travel to MGH for first 2 in patient sessions after screening, consent, baseline and randomization.	5. Allow caregivers to participate in hospital visit via video when caregivers work full time or live out of state. Accommodate evening hours.
6. Low internal consistency on measures with reversed scored items for patients at baseline.	6. Administer Mini Mental Status Exam to confirm cognitive ability to participate and answer study questionnaires. Check patients’ answers immediately after baseline questionnaire completion, assist with reversed scored items.

With funding from the National Institute of Nursing Research, we are now conducting a proof of concept RCT that enrolls at risk dyads with any acute brain injury, as our prior studies did not find any differences in emotional distress by type of diagnoses [7–10, 15]. This RCT also employs an attention placebo control matched in time and dose with RT, in order to strengthen the internal validity of findings. That is directly informed by lessons learned from the current study (Table 5). This RCT is directly informed by lessons learned from the current trial. Specifically, we have strengthened our collaboration with the nursing team in the Neuro-ICU, so that nurses now see the value of our work and are invested in helping with the study. We engaged nurses in the development and refinement of study procedures including methodology for recruitment and retention, have identified a nurse champion who is the point of contact on all cases, and keep nurses actively involved through monthly presentations and anonymous surveys of engagement and satisfaction.

Our current recruitment strategies are more effective and efficient. Each morning, study staff meets with the nurse champion who identifies all potential participants who are medically and cognitively able to enroll. The nurse champion or bedside nurse introduce the study team to the patient through a “warm hand-off” to increase buy in, coordinate screening of informal caregivers, as well as other services on the floor to ensure uninterrupted time for consent, questionnaire administration, and in-hospital sessions. We are now generally completing enrollment and the first dyadic session in 1 day to minimize drop-off after screening or after consent and before first sessions. We now adopt a more flexible approach to screening patients for emotional distress. While we approach participants as soon as possible after diagnoses, in situations where mental status is low or the medical team does not think the patient is able to consent and meaningfully participate, our team will approach patients at later timepoints prior to discharge. We are also flexible in conducting the in-hospital visits and allow caregivers to participate virtually in cases where the caregiver lives out of state and is unable to come to the clinic during hospital hours, and conduct visits early morning and evenings when needed.

We also considered revisions to study measures in the current proof of concept RCT. Though we observed an unusual pattern of uniform responding (SD = 0) among RT caregivers, our measure of program acceptability has demonstrated utility in treatment studies for a number of chronic medical conditions [58, 59], and was likely due to our small sample size (*n* = 6 caregivers). Further, we observed low internal reliability in some of our baseline questionnaires administered to patients, though most were within or near the ranges of coefficients reported in published studies. Specifically, patients exhibited inconsistent response patterns to the IBM, a measure of the patient-caregiver relationship that assesses dyadic care and dyadic control. While outside of the scope of the present study, it is possible that the circumstances surrounding the hospitalization impacted patient consistency in responding to the questionnaire items. For example, participants may have responded to the prompt that their partner, “Tends to control everything I do,” considering orders from their medical team to promote recovery. Recognizing that the IBM may have conflated expressions of care and concern, we instead included the Dyadic Relationship Scale [63] as a measure of positive dyadic interactions in our current proof of concept RCT. Reliability for the DRS has also been established in cognitively impaired patient populations [64]. Following ours and others observations that some patients’ responses may be impacted by cognitive functioning [65], we are now ensuring the validity of patient measures in the hospital by checking patient’s mental status before administration and by checking answers to the reversed scored items immediately after questionnaire completion. Using these strategies, our ability to recruit participants has dramatically increased, and we have enrolled 52 participants over the course of 8 months.

In sum, the present feasibility randomized control trial provided valuable information for the design of future studies and further development of RT. Using lessons learned from this feasibility trial, we substantially improved methodology and recruitment and are now conducting a proof of concept RCT where we explore treatment effects with a larger sample and pay particular attention to addressing depressive symptoms where these are present. Investment in this proof of concept trial rather than directly conducting a fully powered RCT is allowing us to test proposed methodologies and lessons learned from this trial without the risk wasting time and resources. This is particularly important as prior critical care trials aimed at preventing chronic emotional distress after critical care admission have failed to show efficacy [66].

## Conclusions

We conducted a feasibility trial of Recovering Together, the first dyadic intervention aimed at preventing chronic emotional distress in at-risk stroke patients and their informal caregivers, relative to minimally enhanced usual care (MEUC). We found promising evidence for the feasibility and acceptability of recruitment and study procedures that directly informed a proof of concept feasibility RCT and a future hybrid efficacy-effectiveness trial. We provide information on valuable lessons learned that can inform critical care research for medical patients.

## Data Availability

Original data is stored in a secure database within the Partners’ REDCap system. Scored and cleaned data, as well as output for analyses, are available upon request from the study PI, Ana-Maria Vranceanu.

## Abbreviations

RCT: Randomized controlled trial
RT: Recovering Together
PTS: Post-traumatic stress
Neuro-ICU: Neurosciences intensive care unit
MEUC: Minimally enhanced usual care
CBT: Cognitive behavioral therapy
DBT: Dialectical behavior therapy
HADS: Hospital Depression and Anxiety Scale
MCIDs: Minimally important clinical differences
PCL-C: PTSD Checklist-Civilian Version
DSM: Diagnostic and Statistical Manual for Mental Disorders
GSE: General Self-Efficacy Scale
MOCS-A: Measure of Current Status Part A
CAMS: Cognitive and Affective Mindfulness Scale-Revised
CEQ: Credibility and Expectancy Questionnaire
CSQ-3: Client Satisfaction Questionnaire
PI: Principal investigator
REDCap: Research Electronic Data Capture
